# Endovascular Treatment of “Donut-Shaped” Aneurysm—A Case Series

**DOI:** 10.3390/medicina60071116

**Published:** 2024-07-09

**Authors:** Dragoslav Nestorovic, Igor Nikolic, Ana Stankovic, Mladen Bila, Vladimir Cvetic, Marko Miletic, Vladimir Jovanovic, Goran Tasic

**Affiliations:** 1 Center for Radiology, University Clinical Centre of Serbia, Pasterova 2, 11000 Belgrade, Serbia; drdragoslavnestorovic@gmail.com (D.N.); stankovicana@live.com (A.S.); drvladimircvetic@gmail.com (V.C.); 2 Clinic for Neurosurgery, University Clinical Centre of Serbia, Koste Todorovića 4, 11000 Belgrade, Serbia; i.m.nikolic@gmail.com (I.N.); vladimir.jovanovic1969@gmail.com (V.J.); goran.tasic@med.bg.ac.rs (G.T.); 3 Medical Faculty, University of Belgrade, Dr Subotića Starijeg 8, 11000 Belgrade, Serbia; 4 Clinic for Ophthalmology, University Clinical Centre of Serbia, Pasterova 2, 11000 Belgrade, Serbia; mladen.bila@gmail.com

**Keywords:** intracranial aneurysm, donut aneurysm, endovascular, flow-diverter stent, embolization

## Abstract

*Background and Objectives*: Partially thrombosed aneurysms represent a subset primarily found within large and giant aneurysms. The presence of an intraluminal thrombus can cause an aneurysm to present in different shapes upon angiographic examination. We present a series of five cases of “donut-shaped” aneurysms observed over the past decade at the Clinic for Neurosurgery in the University Clinical Centre of Serbia. *Materials and Methods*: The management of “donut-shaped” aneurysms was accomplished through endovascular interventions, employing techniques such as the deployment of flow-diverting stents or a combination of stent placement and coil embolization. *Results*: Four out of five patients underwent endovascular treatment, yielding positive outcomes with complete thrombosis of the aneurysms during follow-up. The fifth patient was successfully diagnosed; however, due to their deteriorating condition, treatment was not feasible. *Conclusions*: Given the potential life-threatening complications associated with this entity, accurate diagnosis and appropriate management are crucial. In our cohort, endovascular interventions demonstrated efficacy in the majority of cases, underscoring the significance of this approach in treating “donut-shaped” aneurysms. Nevertheless, considering the rarity of this condition, further research is justified to refine diagnostic and therapeutic strategies for these complex intracranial vascular anomalies.

## 1. Introduction

Partially thrombosed aneurysms represent a unique subset primarily observed within the spectrum of large and giant aneurysms [1]. These occurrences are documented in a significant proportion, ranging from 17.5% to 33%, among cases involving giant aneurysms [2]. The presence of an intraluminal thrombus within these aneurysms introduces a complexity that often manifests in diverse morphological presentations seen upon angiographic examination [3].

The term “donut-shaped” aneurysm characterizes a distinct subset of partially thrombosed aneurysms, distinguished by their particular morphological features resulting in a ring (donut)-like appearance. This radiological manifestation is exceptionally rare, with only four documented cases identified within the past decade at our clinic. Since the seminal Italian giant aneurysm study in 1988, there has been low attention devoted to exploring this intriguing phenomenon [4]. We hereby present five cases of “donut-shaped” aneurysms documented in the past several years, which were admitted to the Clinic for Neurosurgery in the University Clinical Centre of Serbia (Table 1).

## 2. Case Report

### 2.1. Case 1

**Figure 1:**
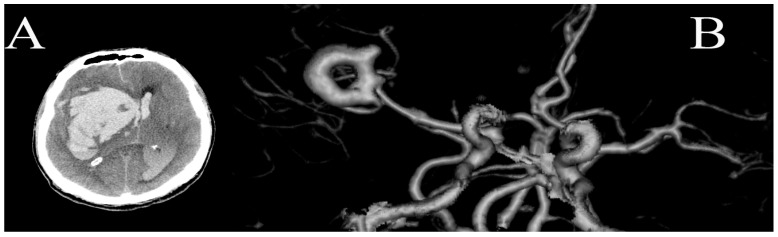
(**A**) Non-enhanced CT demonstrates subarachnoid and extensive intraparenchymal hemorrhage with propagation into the ventricular system; (**B**) CT angiography showed donut-shaped aneurysm of the right middle cerebral artery bifurcation.

A 59-year-old female patient was referred to the Emergency Center for evaluation following a loss of consciousness. Computed tomography (CT) imaging revealed a significant intracerebral hemorrhage (ICH) with extension into the ventricular system. Subsequent computed tomography angiography (CTA) revealed a substantial saccular middle cerebral artery (MCA) aneurysm with central thrombosis, imparting an atypical donut shape to the aneurysm (Figure 1). Upon admission, the patient presented with a significantly diminished Glasgow Coma Scale (GCS) score of five, indicative of a severe neurological compromise. Unfortunately, the patient’s condition rapidly deteriorated, culminating in death two days following admission.

### 2.2. Case 2

**Figure 2:**
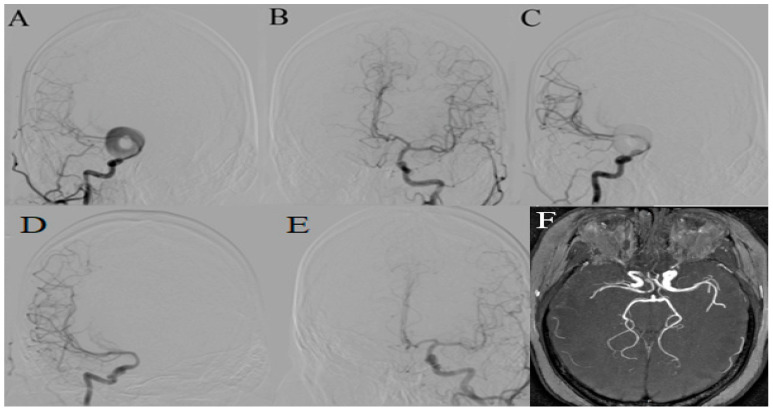
DSA (**A**) AP projection demonstrating a donut-shaped aneurysm localated at the C6 and C7 segments of the right ICA; (**B**) AP projection of the left ICA revealing an elevated A1 segment of left ACA; (**C**) AP projection of the right ICA after the flow-diverter stent placement, revealing prompt contrast stagnation; (**D**) Follow-up DSA after 6 months, right ICA frontal view, demonstrating occlusion of the aneurysm; (**E**) Follow-up DSA, left ICA, with the left A1 segment of ACA “returning” to its normal position; (**F**) Follow-up MRA after 18 months, thick slices revealing no aneurysm remnant and preserved patency of the right ICA.

A 33-year-old female patient was admitted to the Clinic for Neurosurgery in the University Clinical Centre of Serbia with persistent headaches and bilateral visual impairment, notably more pronounced on the left side. Magnetic Resonance Angiography (MRA) revealed a 26.5 mm × 25 mm donut-shaped aneurysm on the right C6 segment of internal carotid artery (ICA) with a neck measuring 11 mm. A pre-aneurysmatic narrowing of the proximal ICA was noted, measuring 3.2 mm proximally and 1.9 mm in the pre-aneurysmatic part. The right A1 segment of anterior cerebral artery (ACA) was aplastic in appearance, and the right ACA was filling from the left A1 segment, which was elevated due to compressive effect of the aneurysm. A flow-diverting (FD) stent was deployed, resulting in initial stagnation of contrast agent within the aneurysm sac (O’Kelly-Marotta (OKM) grade B3). At the 6-month digital subtraction angiography (DSA) follow-up, the aneurysm appeared completely thrombosed, accompanied by a restoration of the contra-lateral A1 segment of ACA to its anatomically expected position and improved visual status. Subsequent 18-month MRA reaffirmed the positive outcome of treatment (Figure 2).

### 2.3. Case 3

**Figure 3:**
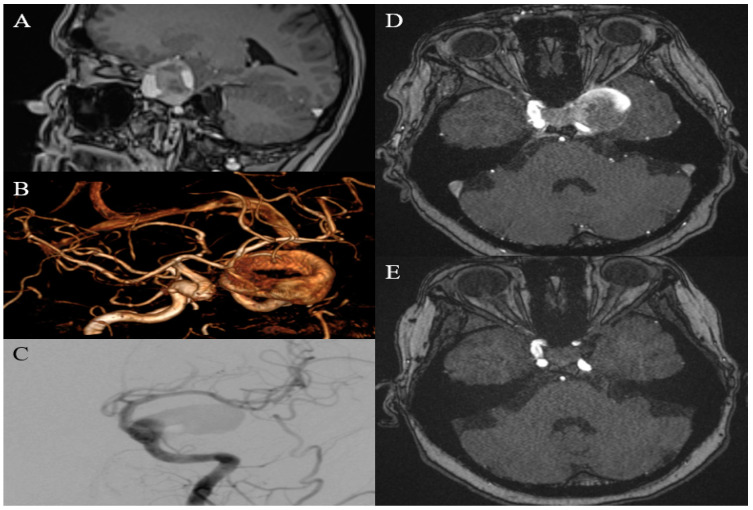
(**A**) MRI revealed a giant, partially thrombosed aneurysm within the cavernous segment of the left ICA with circular flow and “apple core” shaped thrombus in the aneurysmal sac; (**B**) MRI–TOF-3D reconstruction shows donut shape of the aneurysm; (**C**) DSA following the flow-diverter implantation, AP projection; (**D**) MRI before endovascular intervention; (**E**) Follow-up MRI after 12 months.

A 51-year-old female patient presented with complaints of vision loss in her left eye, frequent headaches, nausea, and weakness in her left arm. An MRI revealed a giant aneurysm measuring 36 mm × 30 mm on the intracranial segment of the left ICA, characterized by a donut-shaped formation on the Time of Flight (TOF-3D) images. Given the aneurysm’s substantial size and location engulfing segments C4–C6 of the ICA, an FD stent was deployed across its broad neck. An immediate post-implantation assessment indicated an OKM grade B3. The follow-up MRI at 12 months demonstrated a reduction in aneurysm size and complete exclusion from circulation (Figure 3).

### 2.4. Case 4

**Figure 4:**
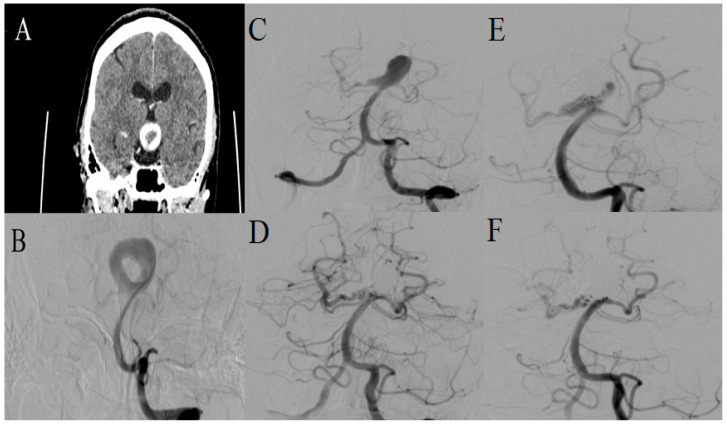
(**A**) Contrast-enhanced CT identified a ring-shaped wide-necked aneurysm at the basilar tip; (**B**) DSA of the posterior circulation displaying a giant partially thrombosed donut-shaped aneurysm at the basilar tip. (**C**) Preprocedural DSA following a treatment delay demonstrated significant thrombosis within the majority of the aneurysm; (**D**) Postprocedural DSA after placement of the stent over aneurysm neck and subsequent coiling; (**E**) DSA on 6-month follow-up revealed coil impaction within the thrombus with residual aneurysm presence; (**F**) Postprocedural DSA following the coiling procedure.

A 48-year-old female patient incidentally presented with a giant donut-shaped aneurysm at the basilar tip during a CT examination of the paranasal sinuses. Initially, a neuroradiologist suspected an expansive process, prompting a contrast-enhanced CT scan which revealed a vascular etiology of the anomaly. Subsequent DSA indicated a 25 mm radius for the aneurysm with a 14.5 mm neck diameter (Figure 4). Due to a contrast allergy, the embolization procedure was delayed by six weeks following the appropriate preparation. Pre-procedural angiograms revealed the occlusion of the entire right side and a portion of the left “wing” of the aneurysm. A stent was placed across the neck in her left posterior cerebral artery (PCA), followed by coiling of the aneurysm. The patient was discharged 14 days later in good general condition, only with complaints due to allergy-related issues. On the 6-month follow-up, DSA revealed an aneurysm remnant measuring 19 × 11 mm due to coil impaction within the thrombus mass. The residual portion was addressed with an additional coil embolization during the same procedure, excluding the aneurysm once again. Upon discharge, the patient had the same complaints regarding allergy symptoms, expressing higher discomfort compared to previous occurrence. Taking the patient’s specific condition into account, she refused the contrast allergy premedication protocol and CTA, requesting an MRA follow-up. Six months after the intervention, two residual segments of the aneurysm were identified, measuring 5.5 mm × 4 mm on the right and 7 mm × 3.5 mm on the left side, which eventually morphed into the single residuum upon a 13-month follow-up diagnostic assessment. Throughout this interval, the patient remained asymptomatic, refusing further intervention until the onset of aneurysm-related symptoms.

### 2.5. Case 5

**Figure 5:**
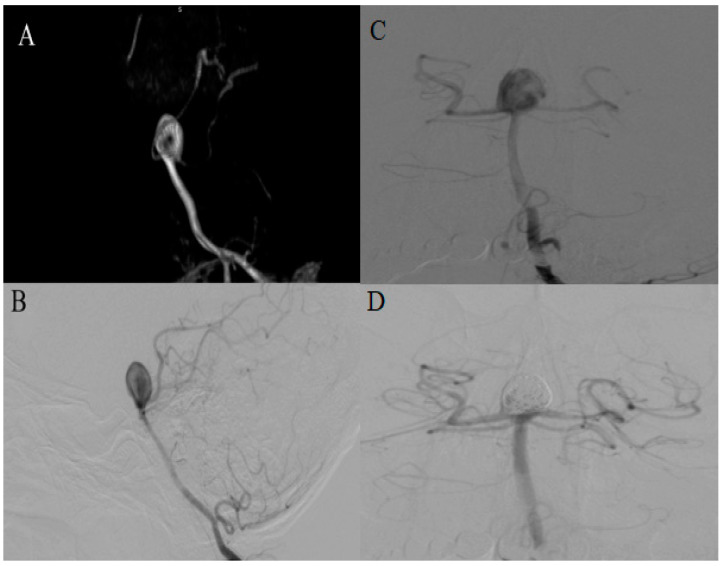
(**A**) MRI-TOF-3D reconstruction; (**B**) Preprocedural DSA of the posterior circulation in the lateral projection highlighting the morphological transformation of the initially donut-shaped aneurysm after DAPT preparation due to partial dissolution of central thrombus; (**C**) preprocedural DSA in the AP projection; (**D**) Postprocedural DSA after implantation of the pCONus2 device and coiling.

Our last patient in this series involves a 46-year-old male with a donut-shaped aneurysm at the tip of the basilar artery. The aneurysm was incidentally detected during an MRI conducted as part of a routine medical examination, reporting a mild headache as the only symptom. Measuring 12.5 mm × 10.5 mm, the aneurysm exhibited a characteristic donut-shape configuration. Its main channel extended back to the aneurysm neck, while the right channel terminated as the right PCA. Two weeks following the discovery, the patient underwent endovascular treatment. Given the aneurysm’s wide neck, the patient received dual antiplatelet therapy (DAPT) in preparation for the pCONus2 device deployment. Pre-procedural DSA revealed alterations in the aneurysm presentation with partial dissolution of the mid-positioned thrombus. Subsequently, during the procedure, the device was positioned to cover the aneurysm neck, followed by coil embolization of its sac (Figure 5).

## 3. Discussion

A partially thrombosed cerebral aneurysm presenting in a donut-shaped configuration represents an exceedingly rare occurrence, with only a handful of documented cases in the existing literature [3,4,5,6,7,8,9,10,11,12,13,14]. The primary method of management has typically involved endovascular interventions, yet these interventions have resulted in a challenging prognosis marked by a high likelihood of relapse.

Characterized by a thrombus formation within the aneurysmal sac suggestive of an “eaten apple core”, the thrombus extends between opposing points on the sac wall, as a result of the peripheral orientation of blood flow. The hemodynamic pattern concentrates blood flow within a circular area along the aneurysm wall, producing a distinctive donut-shaped appearance on angiographic examinations [5].

The formation of thrombus within the aneurysmal sac is a dynamic process, varying in degree. In intracranial aneurysms, the emergence of a donut-shaped aneurysm can be attributed to alterations in flow dynamic conditions. Additionally, such morphology can be disrupted by the progression of thrombosis, as evidenced in our fourth case [6].

The surgical and endovascular approaches for “donut” aneurysms have not significantly differed from that of typical “berry” aneurysms thus far. Van der Schaaf et al. asserted that both surgery and embolization are viable options for patients in good general condition, with embolization being preferred for those in poorer health due to its lesser invasiveness [15]. Considering all aspects, Seifert et al. advocated for an interdisciplinary approach to unruptured aneurysms. They emphasized the importance of carefully selecting patients and collaboratively deciding on the most appropriate treatment modality to achieve the optimal outcome with minimal complications [16]. Hoh’s guidelines for the management of aneurysmal subarachnoid hemorrhage (SAH) also indicated the necessity of an interdisciplinary approach, given that morbidity is influenced by numerous factors [17]. However, in situations where the aneurysm is located in the posterior circulation, treatment options are somewhat limited. In this scenario, the absolute advantage of endovascular treatment and angio-architectonic remodeling is unquestionable. Direct surgery in the posterior circulation requires meticulous conditions and is associated with a notably higher rate of perioperative morbidity (47%) and mortality (23%), whereas endovascular intervention carries an average morbidity risk of 12% and mortality of 4% [18,19]. On the contrary, regarding the endovascular treatment, the use of flow-diverting (FD) stents is discouraged due to a significantly higher risk of mortality, ischemic stroke, and perforator infarction and should therefore be reserved for carefully selected patients only [5,20]. While FD stents are considered preferred approach for large and giant anterior circulation aneurysms, their implantation in posterior circulation cases remains a matter of debate [21].

When addressing aneurysms within the posterior brain circulation, particularly those located at the apex of the basilar artery, challenges persist due to the controversies surrounding the utilization of flow-diverting stents and the necessity for a more traditional approach, often resulting in subsequent recurrences. In our evaluation, the most viable approach for endovascular treatment of posterior circulation aneurysms involves stent-assisted coiling (SAC) or coiling with the aid of aneurysm neck-bridging devices. Furthermore, when opting for coil embolization, it is crucial to recognize that the passable segment of the “donut-shaped” aneurysm has more of a tubular rather than spherical shape, usually demanding the utilization of smaller diameter coils. Implanted coils may be displaced by blood flow and become embedded within the thrombus mass, potentially leading to recanalization of previously occluded portions of the aneurysm. As a result, the presence of intraluminal thrombus may contribute to a five-fold increase in aneurysm recurrence compared to non-thrombosed giant aneurysms treated with coils alone [22].

## 4. Conclusions

This atypical radiological finding of a partially thrombosed intracranial giant aneurysm may be formed due to the specific angulation of concentrated blood flow. Given its rarity and potential life-threatening complications, prompt and appropriate diagnosis and management are imperative.

In our clinical experience, endovascular interventions using flow-diverter stents and stent-assisted coiling have demonstrated efficacy in treating such cases, when diagnosed in a timely manner. However, managing aneurysms in the posterior brain circulation, especially those of the basilar artery, remains a challenge. Debates continue about using flow diverter stents in this arterial region, leading to more frequent utilization of other methods and resulting in frequent recurrences. Given the aforementioned factors, further studies are justified for establishing comprehensive assessment protocols and treatment standards for this rare condition, leading to an improvement in patient outcomes and prognosis.

## Figures and Tables

**Table 1:** Cases characteristics. MCA—middle cerebral artery; ICA—internal carotid artery; BA—basilar artery; L—left; R—right; SAH—subarachnoid hemorrhage; ICH—intracerebral hemorrhage; EVT—endovascular treatment; FD—flow-diverter.

Case	Age	Sex	Diameter	Size	Location	Side	Manifestation	Treatment	Re-treatment	Follow-Up 6 months	Follow-Up 12 months	Follow-Up 24 months	Residual Symptoms
1	59	F	17 mm	Large	MCA bifurcation	L	SAH, ICH	N/A	N/A	N/A	N/A	N/A	N/A
2	33	F	27 mm	Giant	ICA supraclinoid	R	Headache, visual impairment	EVT (FD)	N/A	Complete exclusion	Complete exclusion	Complete exclusion	Can see only shapes on her right eye
3	51	F	36 mm	Giant	ICA infraclinoid	L	Headache, visual impairment	EVT (FD)	N/A	Complete exclusion	Pending	Pending	N/A
4	48	F	25 mm	Giant	BA tip	N/A	Headache, vertigo	EVT (stent + coiling)	Yes (residual volume 19 mm × 9 mm)	Residual volume (7 mm × 5 mm)	Pending	Pending	N/A
5	46	M	14 mm	Large	BA tip	N/A	Headache	EVT (pCONus2 + coiling)	N/A	Pending	Pending	Pending	N/A

## Data Availability

Data is contained within the article.
